# A Novel Case Study of the Use of Real-World Evidence to Support the Registration of an Osteoporosis Product in China

**DOI:** 10.1007/s43441-021-00342-4

**Published:** 2021-10-11

**Authors:** Neal E. Storm, Wen Chang, Tzu-Chieh Lin, Jeff L. Lange, Brian Bradbury, Cathy W. Critchlow, Steven K. Galson

**Affiliations:** 1 Amgen Inc., One Amgen Center Drive, Thousand Oaks, CA USA; 2 Department of Regulatory & Quality Sciences, School of Pharmacy, D.K. Kim International Center for Regulatory Science, University of Southern California, 1540 Alcazar Street, CHP 140, Los Angeles, CA 90089-9014 USA

**Keywords:** Prolia, Denosumab, Real-world evidence, Osteoporosis, Marketing authorization application, China, Regulatory approval

## Abstract

On June 23, 2020, Prolia^®^ (denosumab) was approved by the National Medical Products Administration (NMPA) in the People’s Republic of China as the first monoclonal antibody for the treatment of postmenopausal women with osteoporosis at high risk of fractures. Its brand name in Chinese is 普罗力, a transliteration from the English name “Prolia”, which has an implied meaning of “to give strength to everyone”— a suitable name for a potent anti-resorptive therapy. The approval was supported by a novel marketing authorization application (MAA) that included data from Prolia’s global clinical trial program establishing favorable efficacy and safety, augmented by results from a real-world evidence (RWE) study confirming the effectiveness and safety of Prolia in clinical practice within Taiwan and Hong Kong. Key constructs for this registration-quality RWE study included the fit-for-purpose assessment of data quality, methodology and quantitative assessment of potential biases, good practices of study conduct, and reproducibility of results. Using data from clinical practice in Taiwan and Hong Kong to evaluate the benefits versus risks of Prolia treatment in ethnic Chinese women with postmenopausal osteoporosis, the RWE study results for effectiveness were comparable to efficacy demonstrated in the global clinical trial program and results for safety were consistent with the incidence observed in global post-marketing safety studies. While RWE is often used to monitor postmarket safety of drug products, support health insurance coverage decisions, and inform clinicians on real-world use of medicines, it has not been widely used to support regulatory approval for new medicines in lieu of clinical bridging studies in countries where such studies are required. Well-conducted registrational RWE studies can play a pivotal role in complementing the totality of evidence presented in an MAA. The benefits of such an approach include avoiding the collection of additional placebo-controlled trial data in populations where adequate ethnic characterization of efficacy, effectiveness, and safety may already exist from postmarketing sources, and accelerate access for patients to innovative medicines in important regions. Here, we describe a regulatory case study of a novel MAA incorporating RWE that provided important evidence to confirm the benefit:risk of a new drug and facilitated a label expansion to a new patient population.

## A Question of Bridging

Local patient populations for which treatments are intended have not historically been well represented in pivotal multiregional clinical trials (MRCT) that support drug approvals globally. For this reason, certain countries (e.g., China, India, Russia, South Korea, Taiwan, and Vietnam) generally require local ethnic bridging clinical data or sensitivity analyses as part of marketing authorization application (MAA) submissions [1]. Local data can be obtained by enrolling patients in local clinical sites as part of a global MRCT, by conducting an ethnic bridging study, or by conducting a separate local registrational study when necessary. However, the reliance on local bridging data has historically contributed to the “drug-lag”, which is associated with maturing regulatory frameworks in some countries resulting in more pronounced review and approval timelines for both clinical trial and MAA reviews [1]. Importantly, the logistical challenges of these requirements have historically delayed access to new drugs in these markets, often by years, if there is no feasible method to address local data requirements within the global MRCT. For example, the conduct of placebo-controlled studies can prove challenging from an ethical perspective for products with proven efficacy. An unintended outcome can be reduced quality of life for patients in certain geographies with limited to no access to important new therapies.

As the demographics of China become more urban and older, osteoporosis has come into focus as a serious disease of increasing clinical significance [2]. Hence, there is an identifiable medical need for additional treatment options for Chinese women with osteoporosis [2]. Prolia^®^ (denosumab, 60 mg/mL every 6 months [Q6M]) is a fully human monoclonal antibody that binds and neutralizes RANK ligand (RANKL). Inhibiting RANKL in patients with osteoporosis results in reduced osteoclast numbers and function, thereby decreasing bone loss and increasing bone mass and strength in both cortical and trabecular bone [3]. Initial approval for the treatment postmenopausal women with osteoporosis at high risk for fracture was based on 3-year data from a randomized, double-blind, placebo-controlled, phase 3 MRCT [4]. The totality of clinical trial evidence demonstrates that treatment with Prolia significantly reduces the risk of vertebral, nonvertebral, and hip fractures and, in an open-label extension study, continues to increase bone mineral density (BMD) over 10 years at key skeletal sites, without evidence of therapeutic plateau [5]. In addition, the incidence of fractures at the spine, hip, and non-spine sites remained low with continued treatment for up to 10 years [5]. Prolia has since been approved in approximately 80 regions, countries, and administrative districts worldwide, including the regions of Hong Kong, Japan, Korea, and Taiwan in East Asia.

Under the prior regulatory system in China, drugs approved in other countries but not yet approved in China were considered new drugs (category III), and requirements for approval varied depending on whether the drug was locally manufactured or manufactured outside of China. For new drugs not manufactured in China, the path to licensure, or Imported Drug License (IDL) pathway, was lengthy due to the need for official documentation of approval in key reference countries, supplied in the form of Certificates of Pharmaceutical Product (CPP), and were required prior to the submission of a clinical trial authorization (CTA) to support local clinical bridging studies. The regulations at the time acknowledged that local clinical trial data could be obtained from international multi-center clinical trials (IMCT). However, even if there was a suitable regulatory pathway in the mid-2000s to include clinical sites in mainland China in the global MRCT for Prolia, Amgen’s geographical/operational footprint at the time would have made this option unfeasible.

Since its initial approval in 2010 by the FDA and EMA, Prolia has acquired as of September 2019 an estimated postmarketing exposure of > 17 million patient-years. Its favorable benefit:risk profile has been considered highly stable across different treatment landscapes, indications, ethnicities, and geographies. However, the question remained whether its benefit:risk profile remained favorable in a population of ethnic Chinese women with PMO to support licensure in China. Given the large potential size of the PMO population in China and corresponding gravity of the regulatory decision, there was mutual interest between the China Center for Drug Evaluation at the National Medical Products Administration (NMPA-CDE) and Amgen to identify a suitable evidence package to support approval.

In 2011, China’s State Food and Drug Administration (SFDA; prior name for NMPA-CDE) issued guidance for sponsors seeking an indication of PMO—“Considerations for clinical trials evaluating the treatment of postmenopausal women with osteoporosis drugs.” Thus, a regulatory pathway to registration became available in China: if overseas phase 3 studies demonstrating efficacy and safety had been conducted, a confirmatory trial in China would be required to corroborate results [6]. Bone mineral density (BMD) was considered an acceptable surrogate endpoint, rather than requiring the Sponsor to re-evaluate anti-fracture efficacy (e.g., new vertebral fractures), the primary endpoint generally required for global clinical trials supporting an indication of PMO [7]. The NMPA-CDE’s guidance was consistent with regulatory guidance and precedence from other regions: once an initial marketing authorization has been granted for a new drug for the treatment of postmenopausal women with osteoporosis at high risk of fractures, the use of BMD changes for demonstration of non-inferiority/equivalence to a reference product can be used to support new indications, formulations, dose, and route of administration. Conducting an additional clinical bridging trial would delay an important treatment option for Chinese patients at high risk of fracture, and although alternative treatments were already available on the market in China, Prolia represented a convenient treatment option (administered Q6M) with benefits for patients unable to take bisphosphonates, either because of renal insufficiency, contraindication for bisphosphonate therapy, or other limiting side effects.

At the same time that the NMPA-CDE and Amgen sought to resolve the question of what form of bridging information would be needed to support the registration of Prolia in China, the regulatory environment in China was undergoing significant transformation. In October 2017, the General Office of the Chinese Communist Party’s Central Committee and the General Office of China’s State Council jointly announced an opinion on strengthening reforms to the drug and medical device review and approval process to encourage drug and medical device innovation, also known as Opinion No. 42 or the “Innovation Opinion” [8]. The Innovation Opinion, which contained 36 provisions, served as a blueprint for reform and was accompanied by amendments fundamentally changing China’s Drug Administration Law (DAL) and Drug Registration Rules (DRR) [8]. These reforms included new requirements for registering imported drugs, with the intent to “free drug developers of the time and expense of running large Chinese clinical trials of products that have already proven their safety and efficacy overseas” and accelerate their path to patients, with the only caveat being that “companies must cover ethnic differences in their application” [8, 9]. The October 2017 draft guidance provided two key criteria on the use of foreign clinical trial data in an MAA [8]:

- “The data set should at least contain data related to, among other areas, clinical pharmacology, safety, and efficacy of the drug product”; and
- “The safety and efficacy data should contain an analysis showing that the results for a Chinese patient group are consistent with those for the global patient population”

Thus, the requirements for an MAA could in part be fulfilled by clinical trials conducted overseas, which are otherwise in compliance with the relevant requirements for registration in China and the International Council for Harmonisation of Technical Requirements for Pharmaceuticals for Human Use (ICH); however, the residual question of clinical bridging remained [8]. Based on their review of the data, NMPA-CDE could choose to accept, partially accept, or reject the foreign data [8]. Importantly, under the draft guidance there was some degree of discretion left to Sponsors to propose analyses to confirm consistency of treatment effect, leaving the door open for innovative options.

During this period, the twenty first century Cures Act (2016) was being implemented through draft guidance in the United States (US). The themes of the Cures Act were complementary to the new Chinese legislation likewise designed to enhance efficiencies in the drug development process and facilitate the development and review of new innovative medicines for patients. The Cures Act was accompanied by initiatives that introduced the voice of the patient into the drug development process, modernized clinical trials, and—relevantly—formalized and systemically expanded the use of real-world evidence (RWE) to “support the approval of a new indication” or “support or satisfy post-approval requirements” (21st Century Cures Act, Sec. 3022, Real-World Evidence). RWE was broadly defined as “data regarding the usage, or the potential benefits or risks, of a drug derived from sources other than randomized clinical trials.” The FDA has provided further precision to this definition, distinguishing real-world data (RWD) as “data relating to a patient’s health status and/or the delivery of health care routinely collected from a variety of sources” (i.e., electronic health records, medical claims, data from product registries, patient-generated data, and data gathered from other sources that can inform health status), and RWE as the “clinical evidence regarding the usage and potential benefits or risks of a medical product derived from the analysis of RWD” [10]. Examples of RWE use for regulatory decision-making provided by the Cures Act include adding or modifying an indication or population; changing a dose, dose regimen, or route of administration; or adding comparative effectiveness or safety information [10]. Depending on the specific research question, RWE may be used to further facilitate an understanding of the efficacy and safety of a drug in a heterogenous population representative of actual patients seen in clinical practice (outside of those patients who take part in traditional randomized controlled clinical trials).

## Constructing a Totality of Evidence Regulatory Dossier Using RWE

The substantial safety and efficacy data from global clinical trials would be foundational for establishing the benefits and risks of Prolia in a China MAA. In addition to supportive global clinical trial data from > 20,000 subjects, including the phase 3 pivotal fracture study (FREEDOM study) [4] and its open-label extension that followed subjects for an additional 7 years for up to 10 years of Prolia treatment [5], local ethnic bridging studies in East Asian populations had been conducted. The original marketing application for Prolia submitted to the FDA and EMA included 2 studies conducted in Japanese subjects: a single-dose pharmacokinetic study in healthy postmenopausal Japanese women and a randomized, double-blind, placebo-controlled, dose–response study in Japanese women with PMO.[11, 12] Since the original marketing application, two additional studies have been conducted in East Asian populations: a phase 3 registrational study in Japanese men or postmenopausal women with prevalent vertebral fractures [13] and a 6-month, randomized, placebo-controlled study with a 6-month, open-label extension in PMO in Korea [14]. In each of these countries, available data from postmarketing surveillance studies further confirmed Prolia’s safety post-approval. Collectively, this body of data indicates there is no clinically significant inter-ethnic difference in Prolia pharmacokinetics, pharmacodynamics, efficacy, and safety between East Asian (Japanese) and Western populations. It was therefore anticipated that the response to Prolia in a population of Chinese patients would be consistent with that observed in clinical studies to date. However, the global clinical trial evidence was deemed insufficient to prove this because it did not address the specific regulatory requirements of the DAL/DRR to demonstrate that the responses in Chinese patients were consistent with those observed in the global clinical studies. Nevertheless, it was recognized that in some regions with predominantly Chinese populations where Prolia had been approved (Taiwan in 2011, Hong Kong in 2011, Macau in 2010), high-quality healthcare data were available for research. Consequently, Amgen began to explore the use of RWD and RWE to evaluate Prolia benefit:risk in Chinese populations in these regions for extrapolation to mainland Chinese patients.

Patient experience in real-world clinical practice provides an opportunity to evaluate the use, safety, and effectiveness of drugs and devices in populations outside of the clinical study setting and to further understand populations and outcomes typically excluded from clinical study (e.g., patients with multiple comorbidities and long-term clinical outcomes, respectively) [15]. RWD relating to the delivery of health care or patient health status is routinely and increasingly available from electronic medical records (EMRs), administrative claims data, disease and product registries, and patient-generated data outside of clinical settings (e.g., social media and fitness trackers). RWE regarding the use, safety, and effectiveness of treatments can be obtained from RWD through the application of pharmacoepidemiology research methods, as has been described in China and internationally [10, 16].

A fit-for-purpose design of sufficient quality can leverage RWE from health care systems for efficient regulatory decisions under certain circumstances and in the context of the totality of evidence, with appropriate data and analysis methods [10, 17]. The following key constructs were considered to provide the NMPA-CDE with sufficient evidence and confidence in the methods to enable regulators to assess whether a proposed RWE study would yield valid conclusions [10, 17]:

- Develop a discrete research question that is clinically meaningful and tests a specific hypothesis, understanding the context in which the treatments are used;
- Evaluate if the available data are adequate for describing exposures, outcomes, and covariates;
- Use an appropriate study design to assess treatment effects on clinically meaningful outcomes and evaluate comparability between groups based on prognostic differences and their potential impact on study interpretation;
- Ensure transparency of methods and analysis through proactive stakeholder engagement; and
- Ensure reproducibility of results through appropriate study design methods.

In the case of Prolia, a real-world study was envisioned to answer two discrete regulatory questions of interest from the NMPA-CDE;

1. To evaluate the effectiveness of Prolia for the reduction of clinical osteoporotic fractures among Chinese women with PMO; and
2. To evaluate the safety of Prolia among Chinese women with PMO in Taiwan and Hong Kong.

To answer these questions, we worked with scientists and analysts from the Institute of Clinical Pharmacy and Pharmaceutical Sciences at Cheng Kung University in Taiwan and The Centre for Safe Medication Practice and Research within the Department of Pharmacology and Pharmacy at the University of Hong Kong to conduct a retrospective cohort study. In Taiwan, we used a population-level, claims database (Health Insurance Research Database) that includes demographic and clinical information on diagnoses and procedures for 99.9% of the population in Taiwan [18]. In Hong Kong, we similarly used a population-level, clinical database (Clinical Data Analysis and Reporting System) that includes demographic and clinical information on diagnoses and procedures for all public healthcare services, which include ~ 80% of hospital admissions in Hong Kong [19]. Combined, these data sources comprised a large sample size of > 40,000 Prolia patients and provided longitudinal follow-up data on patients’ receipt of clinical care. The process of using/accessing these administrative data sources and generating the study results at the data analytical centers have been submitted for publication [20]. In brief, for both data sources, only researchers in academia may apply for data access (for a fee), and local data privacy laws and regulations apply. The study protocol was submitted for ethics review and approved by each academic institution [National Cheng Kung University Institutional Review Board (HREC#107-008) in Taiwan and the Institutional Review Board of the University of Hong Kong/Hospital Authority Hong Kong West Cluster (UW 19-154) in Hong Kong]. In neither case could the raw data be removed from the respective data analytical centers [20].

To address the first question relating to effectiveness of Prolia in reducing clinical fractures among Chinese women with PMO, the risk reductions assessed in the real-world study of ethnic Chinese women on Prolia were put in context with the results from the phase 3 MRCT study (FREEDOM). In FREEDOM, Prolia treatment for 36 months reduced clinical vertebral fractures by 68%, hip fractures by 40%, and nonvertebral fractures by 20% relative to placebo [4]. In the Taiwan / Hong Kong real-world study, fracture risks were compared between two cohorts of patients initiating denosumab: treatment cohort (patients administered Prolia 60 mg subcutaneously [SC] every 6 months for up to 10 doses) versus non-treatment cohort (patients discontinuing after 1 dose, which has no known clinical benefit). Additional substudies were undertaken to assess data quality of the primary endpoint of hip fracture and to assess the role of unmeasured confounders. The relative risk reductions for hip fracture, clinical vertebral fracture, and non-vertebral fractures for the treatment cohort versus the non-treatment cohort observed were similar to the reductions in fracture risk demonstrated in FREEDOM [4, 20].

To address the second question relating to safety of Prolia in Chinese women with PMO, we assessed the magnitude of three important identified risks for Prolia (osteonecrosis of the jaw [ONJ], atypical femoral fracture [AFF], and hypocalcemia) in a large longitudinal sample of Chinese patients in the Taiwan and Hong Kong data sources. Of these three risks, the incidence of AFF has been reported to be potentially higher in Asian patients compared with Western patients [21] and was considered of particular interest to evaluate in the Taiwan / Hong Kong real-world study. The incidence rates for ONJ, AFF, and hypocalcemia in Chinese women with PMO living in Taiwan and Hong Kong were within the same range [20] as those observed using similar methodology in other observational data sources in the US (Medicare and United HealthCare/Optum) and the national health registries of Scandinavian countries (Denmark, Norway, and Sweden), as assessed in an ongoing prospective, open-cohort postmarketing observational study (Study 20090522) assessing the long-term safety of Prolia (conducted as a postmarketing commitment with the FDA and the EMA) [22]. Similarly, a prior analysis of a Taiwan osteoporosis population treated with alendronate, raloxifene, or calcitonin had demonstrated an observed incidence of 6.9 to 8.2 ONJ cases per 10,000 person-years [23]. These findings suggest that while there are differences in patient populations, clinical practices, and healthcare systems, the overall rates of ONJ, AFF, and hypocalcemia observed in Chinese patients are relatively low and similar to rates in the Western observational data sources (within the range of ± 1 per 1000).

The results in Chinese women living in Taiwan and Hong Kong [20] support the conclusion that the response to Prolia at a dose of 60 mg SC Q6M in a patient population in China would be consistent with populations in other regions, as observed in the global phase 3 MRCT study (FREEDOM) [4], which included subjects from Western Europe, Eastern Europe, Latin America, and North America. These results bring additional relevant and timely clinical practice-based benefit:risk information supporting the effectiveness and safety of Prolia in Chinese patients, which are comparable to the outcomes from FREEDOM and observations from postmarketing experience with Prolia. This additional evidence appeared well-suited to provide the substantial evidence needed to resolve the residual question on the benefit:risk of Prolia treatment in mainland Chinese women with PMO at high risk of fracture.

## Regulatory Interaction Process

Because of the potential heterogeneity in the quality and rigor of observational trials, it was important to align with the NMPA-CDE on what “good” RWE looked like to develop regulatory-grade evidence to support NMPA-CDE decision-making. Close communication with NMPA-CDE was considered essential at key junctures throughout the regulatory interaction process. Following an initial Communication Meeting with the NMPA-CDE to determine if a RWE-based approach was acceptable to inform whether the benefit:risk of Prolia in Chinese women with PMO was similar to that observed in other populations, the NMPA-CDE advised that experts within mainland China should be consulted in an advisory capacity on behalf of the China scientific community on technical issues relating to protocol development. This recommendation is consistent with good practices for transparency in RWD study design and execution [17], which describes the need to engage multiple key stakeholders (university researchers, clinicians, regulatory agencies) during study design. All experts engaged offered rich opportunities for mutual learning and knowledge sharing, and perspectives on a range of technical questions such as the validity of extrapolating results from Taiwan and Hong Kong to a broader patient population in mainland China and the methods to ensure data quality (e.g., causal inference techniques to ensure data reliability and methods to reduce uncertainty and bias). The process was aided by robust feasibility assessments that ensured the proposed real-world methodology was consistent with good practice. Importantly, these assessments had already been conducted and published prior to initiation of the regulatory interactions with the NMPA-CDE to further increase confidence in the proposed methods [23, 24].

Key areas of NMPA-CDE interest included the proposed methods to control unmeasured confounding and handling of missing data (i.e., primarily BMD). The NMPA-CDE also recommended implementation of pre-specified sensitivity analyses and use of propensity score matching for the primary analysis. Importantly, a second Communication Meeting was held with the NMPA-CDE, at which the protocol was agreed upon with NMPA-CDE prior to initiation. Additionally, the protocol and analysis plan were posted before study initiation on the public study registration site for observational studies at the European Network of Centers for Pharmacoepidemiology and Pharmacovigilance (ENCePP^®^), which is a network coordinated by the EMA for full transparency within the global scientific community (http://www.encepp.eu/encepp/viewResource.htm?id=37411). Once the study was completed and results were available, a Pre-MAA Communication Meeting was held with NMPA-CDE to further discuss the results in the context of global data and how to integrate the RWE and clinical trial data into the MAA. A final issue remained, as it is considered a basic regulatory requirement that patient-level study data be accessible to the scientific community and results reproducible by a regulatory authority. However, laws meant to ensure confidentiality and patient privacy can limit the ability of drug sponsors to submit datasets along with regulatory submissions–a challenge associated with the use of RWE in regulatory submissions that is not unique to any one geography. The Joint International Society for Pharmacoepidemiology-Professional Society for Health Economics and Outcomes Research (ISPE-ISPOR) Special Task Force on Real-World Evidence in Health Care Decision Making[25] offered two practical solutions based on concepts of reproducibility:

1. Direct Replication: a reproducible study is one for which investigators implementing the same methods in the same data can obtain results with the same clinical interpretation; and
2. Conceptual Replication: a reproducible finding is one for which the same question is addressed with different data and operational procedures.

Direct replication dictates that if independent investigators applied the same operational choices to the same longitudinal data source, they should be able to obtain the same results (or a near-exact reproduction) as the original analysis. For a regulatory authority to replicate results, an option was proposed for a government-designated third party (e.g., local opinion leader or academic) and/or special non-governmental employee acting on behalf of the NMPA-CDE to potentially reproduce study results, if deemed necessary. In addition, the concern was addressed in the marketing application by submitting a new submission component: a “Technical Review and Analysis Reproducibility Plan” (TRARP). This document is a special reviewer guide that provides alternative strategies to reproduce results, in lieu of actual real-world datasets in the submission, and provides all analytical components needed for direct replication of results. Thus, every method used to analyze the results was transparently shared with the NMPA-CDE. The TRARP included the following:

- Data structure of the raw data source;
- Review of the data management process from raw data source to analytical dataset;
- Structure and variable lists for analytical datasets;
- Clear natural language description for SAS coding for key analyses; and
- Detailed study protocol and statistical analysis plan.

Conceptual replication (i.e., to perform a study in two different data sources by two different research institutions to facilitate an assessment of reproducibility of the results) was operationally addressed under a single protocol. In doing so, parallel analyses allowing qualitative comparison between RWE derived from datasets in Taiwan and Hong Kong were performed to ensure reproducibility of findings and submitted in the MAA.

## Discussion

Although the use of RWD/RWE to improve the efficiencies of clinical trials is well established and encouraged by major regulatory authorities, there are potential regulatory use cases for RWE beyond those described in available guidance and regulatory precedence. The range of potential uses for RWD/RWE in regulatory decision-making is still evolving and expanding. As has been suggested by Bolislis et al., although “the current application of RWD has been limited to specific cases, there is a potential to further explore and develop its application” [26]. Industry plays an obligatory role in the process of identifying and bringing forward scientifically robust RWE use cases to health authorities for evaluation in appropriate regulatory situations, and the publication of these use cases is critical to advancing the use of RWE in regulatory decision-making by building confidence in the validity of such evidence. In addition, drug developers play a key role in ensuring the scientific robustness of these proposals to ensure the quality of the data upon which regulatory decisions are based, the reproducibility of results as required by regulatory authorities, and the transparent communication of methods for the scientific community to validate.

The basic framework set forth by Berger et al. for assessing when RWD/RWE is “fit-for-purpose” to inform regulatory decision-making offers concepts generalizable to this situation. The principles used in this case of regulatory decision-making are reflected in the NMPA-CDE’s *Guideline of Using Real-World Evidence to Support Drug Research & Development and Evaluation (Interim)* [27]. Key features of the Prolia case, provided as an example when RWE/RWD can be considered appropriate to support regulatory decisions, were described as follows:

- “The study followed the good practice for real-world research, and the study protocol had been disclosed in advance.”
- “The RWD source was well representative of the study population, and the sample size reached up to more than 40,000 subjects.”
- “The primary endpoint of the study was verified by medical record review; propensity score matching was used as the primary analysis method, multiple methods including inverse probability of treatment weighting and high-dimensional propensity score adjustment were used for sensitivity analysis, and the impact of unmeasured confounders were quantitatively assessed.”
- “Results of the real-world study were similar to those of the global RCT study and reproduced with real-world data from different data sources and different research institutions.”

A process of early, transparent, and step-wise interactions with NMPA-CDE reviewers and their advisors in the China scientific community were beneficial to align on the innovative study design and the data package provided in the MAA submission required to inform NMPA-CDE’s evaluation of the benefit:risk of Prolia. Prolia’s approval by the NMPA-CDE on June 23, 2020 is testament to the effectiveness of the transformative drug regulatory reforms occurring in China since 2015, which sought to: (1) solve the problem of drug approval lag and backlog of submissions; (2) improve the quality of drug evaluation and approval; (3) encourage the development of innovative new drugs; and (4) improve the transparency of drug evaluation and approval. This substrate of rapid regulatory reform and a common interest in helping patients in China created an environment of collaborative scientific exchange among the stakeholders, most importantly the NMPA-CDE, that was conducive to innovation, ultimately improving the quality of our RWE study designs and methodology. Further development of RWE methodology, coupled with global regulatory reforms and willingness by companies to propose novel regulatory strategies that incorporate the use of RWE, could result in therapies becoming more rapidly available in many countries. These advances may eventually reduce the need to randomize patients with serious disease to placebo treatments when the evidence for active drug effectiveness has been adequately demonstrated. Thus, while RWD/RWE use cases that support regulatory decision-making are accumulating, the approval of Prolia in the People’s Republic of China may offer a novel solution for drug products in similar situations where additional efficacy and safety data (in this case, ethnic bridging) are required by a health authority as a pre-condition for approval.
